# Getting a Vaccine, Jab, or Vax Is More Than a Regular Expression. Comment on “COVID-19 Vaccine-Related Discussion on Twitter: Topic Modeling and Sentiment Analysis”

**DOI:** 10.2196/31978

**Published:** 2022-02-23

**Authors:** Jack Alexander Cummins

**Affiliations:** 1 Manchester Essex Regional High School Manchester, MA United States

**Keywords:** COVID-19, vaccine, vaccination, Twitter, infodemiology, infoveillance, topic, sentiment, opinion, discussion, communication, social media, perception, concern, emotion, natural language processing

Lyu et al [1] used natural language processing techniques to analyze the topics and sentiment of Twitter conversations related to the COVID-19 vaccine using the Twitter chatter data set created by Georgia State University’s Panacea Lab. Tweets that contained any of the following keywords “vaccination,” “vaccinations,” “vaccine,” “vaccines,” “immunization,” “vaccinate,” and “vaccinated” were selected for further analysis (eg, topic modeling and sentiment analysis).

I would suggest that the study might be enhanced by including additional keyword searches for vaccination synonyms identified by a method such as the Continuous Bag of Words (CBOW) word2vec model [2]. While the study focused on formal language such as “vaccination” and “vaccine,” other colloquial terms are commonly used on Twitter and other social media platforms to describe the vaccination process.

I identified synonyms for “vaccination” commonly used on Twitter by using the *gensim* implementation of the CBOW word2vec model [3]. This model predicts synonyms and related words by creating vector representations of words. Words with similar vector representations are more likely to be synonyms than words with dissimilar vector representations. I trained the CBOW word2vec model on 503,862 tweets containing the keyword “covid” or “corona” from June 24-27, 2021, collected through the *rtweet* package [4]. The keyword pattern search results included tweets using words related to COVID-19 such as “covid-19” or “coronavirus.”

Out of the 503,862 COVID-19–related tweets downloaded with *rtweet*, 94,768 contained at least one of the words searched for in the study by Lyu et al [1]. In addition, a total of 22,587 tweets used the terms “shot,” “shots,” “jab,” “jabs,” “jabbed,” “vax,” or “vaxxed.” The words “shot” or “shots” were used in 9017 tweets. The words “jab,” “jabs,” or “jabbed” were used in 7021 tweets. The words “vax” or “vaxxed” were used in 4081 tweets. Out of the 22,587 tweets that contained these alternative terms, 15,855 (70.2%) were tweeted by users who self-disclosed their location on their user profile. Using the Nominatim application programming interface, it was possible to identify geocoded location, including country, for 13,101 of the 15,855 user-disclosed locations [5]. Of these 13,101 geocoded tweets, 3111 were from the United Kingdom, of which 2261 used “jab,” “jabbed,” or “jabs.” Among the geocoded tweets, 4910 were from the United States; of these, 2704 included “shot” or “shots” and 1130 used “vax” and “vaxxed.”

I would propose that researchers performing keyword searches on social media chatter consider using the CBOW word2vec model to enhance their studies by expanding the number of comments they capture and to reduce geographic or population bias that may occur from the preselection of terminology. The CBOW word2vec model can help capture more completely the full range of word choices used by social media users.

## Abbreviations

CBOW: Continuous Bag of Words
